# Editorial: Metabolic cell death in cancer: innovative therapeutic avenues for cancer treatment

**DOI:** 10.3389/fonc.2026.1827049

**Published:** 2026-06-09

**Authors:** Zhou Yang, Guang Lei, Yize Li

**Affiliations:** 1 Department of Medical Oncology, Shanghai East Hospital, Tongji University School of Medicine, Shanghai, China; 2 Department of Experimental Radiation Oncology, The University of Texas MD Anderson Cancer Center, Houston, TX, United States; 3 Department of Medicine, McDonnell Genome Institute, Washington University in St. Louis, St. Louis, MO, United States

**Keywords:** apoptosis, cancer vulnerability, cuproptosis, ferroptosis, hypoxia, metabolic reprogramming, regulated cell death, therapeutic targeting

Metabolic reprogramming is now widely recognized as a defining hallmark of cancer that extends far beyond its classical role in sustaining uncontrolled proliferation. By reshaping nutrient utilization, redox balance, and mitochondrial function, tumor cells actively modulate their stress tolerance and survival capacity within hostile microenvironments. Increasing evidence indicates that these metabolic adaptations also fundamentally determine cellular susceptibility to diverse forms of regulated cell death. Apoptosis, ferroptosis, cuproptosis, and the recently characterized disulfidptosis are no longer viewed as isolated execution pathways, but rather as processes tightly governed by metabolic state. The Research Topic “Metabolic Cell Death in Cancer: Innovative Therapeutic Avenues for Cancer Treatment” brings together five complementary studies that collectively advance a unifying concept: the same metabolic plasticity that enables tumor survival also generates context-dependent vulnerabilities that can be therapeutically exploited.

## Metabolic rewiring as both an advantage and a liability

Amino acid metabolism exemplifies how cancer cells finely balance biosynthetic demands with survival under metabolic stress. The contribution focusing on serine metabolism provides a systematic overview of how tumors reprogram serine synthesis and uptake to support one-carbon metabolism, nucleotide biosynthesis, redox homeostasis, and epigenetic regulation. Beyond its anabolic functions, serine availability critically influences intracellular redox buffering capacity and resistance to oxidative stress. Importantly, this work (Liu et al.) highlights that serine dependency represents a metabolic liability rather than a neutral adaptation. Therapeutic strategies based on serine deprivation therefore illustrate a broader principle: targeting essential metabolic nodes can destabilize tumor homeostasis and lower the threshold for regulated cell death, particularly in tumors that are highly dependent on specific nutrient pathways.

## Signaling–metabolism integration determines apoptotic competence

Metabolic regulation of cell death is inseparable from oncogenic signaling networks that integrate growth cues with survival decisions. The study (Zhang et al.) demonstrating that Cetrorelix promotes apoptosis in epithelial ovarian cancer through suppression of the PI3K–AKT pathway and activation of FOXO1 underscores this integration. Rather than functioning solely as a growth-inhibitory intervention, modulation of this signaling axis reactivates intrinsic apoptotic programs that are frequently suppressed during tumor progression. These findings reinforce the notion that therapeutic efficacy often depends on restoring apoptotic competence, rather than merely inhibiting proliferation.

## Hypoxia as a central checkpoint of metabolic cell death resistance

Hypoxia remains a pervasive feature of solid tumors and represents a major driver of metabolic adaptation and therapeutic resistance. The study (Song et al.) showing that Cucurbitacin B suppresses non-small cell lung cancer progression through inhibition of HIF-1α via ZFP91 adds important mechanistic insight into hypoxia-driven metabolic rewiring. Beyond its canonical roles in promoting glycolysis, angiogenesis, and survival under low-oxygen conditions, HIF-1α has emerged as a critical regulator of cell death susceptibility. Notably, recent evidence demonstrates that HIF-1α actively drives cancer resistance to cuproptosis by reshaping mitochondrial metabolism and copper utilization, thereby protecting tumor cells from copper-dependent proteotoxic stress (1). These observations position hypoxic signaling as a central gatekeeper linking metabolic adaptation to resistance against emerging metabolism-driven cell death modalities.

## Genetic context constrains metabolic vulnerabilities

The regulation of metabolic cell death is highly context dependent and shaped by tumor lineage and genetic architecture. The case series and literature review describing neuroepithelial tumors of the central nervous system harboring EWSR1::PATZ1 fusion expands the molecular classification of these rare entities (Feldmann et al.). While direct connections to metabolic cell death mechanisms remain to be fully defined, precise molecular characterization provides an essential framework for future studies aimed at uncovering lineage-specific metabolic constraints. Such genetically defined tumor subtypes may exhibit unique metabolic dependencies that could be leveraged to induce selective cell death (2).

## Ferroptosis as an emerging therapeutic opportunity

Among non-apoptotic forms of regulated cell death, ferroptosis has gained substantial attention due to its intimate association with iron metabolism, lipid peroxidation, and redox control. The identification of TIMP1 as a regulator of ferroptosis sensitivity in colorectal cancer, supported by integrated bioinformatic and experimental approaches, highlights the influence of extracellular matrix–associated factors on ferroptotic vulnerability (Tang et al.). Together, ferroptosis and cuproptosis exemplify a paradigm shift in cancer cell death research, in which mitochondrial activity, redox balance, and metal homeostasis emerge as central determinants of cell fate rather than downstream consequences of stress. This conceptual framework further strengthens the rationale for targeting ferroptosis in tumors that are refractory to conventional apoptosis-based therapies.

## Concluding remarks and future perspectives

Taken together, the studies assembled in this Research Topic support a conceptual redefinition of metabolic cell death—not as a terminal outcome of metabolic stress, but as a regulatable vulnerability actively sculpted by tumor evolution and microenvironmental adaptation. Therapeutic strategies that effectively exploit these mechanisms will likely require rational combinations that simultaneously disrupt metabolic adaptation and reactivate cell death pathways. As emerging modalities such as cuproptosis continue to be mechanistically defined, integrating metabolic profiling with cell death phenotyping and microenvironmental analysis will be essential. Ultimately, converting cancer’s metabolic flexibility into a therapeutic liability represents a promising path toward more durable and precise anticancer interventions.
